# *SIGMAR1* mutation associated with autosomal recessive Silver-like syndrome

**DOI:** 10.1212/WNL.0000000000003212

**Published:** 2016-10-11

**Authors:** Alejandro Horga, Pedro J. Tomaselli, Michael A. Gonzalez, Matilde Laurà, Francesco Muntoni, Adnan Y. Manzur, Michael G. Hanna, Julian C. Blake, Henry Houlden, Stephan Züchner, Mary M. Reilly

**Affiliations:** From the MRC Centre for Neuromuscular Diseases (A.H., P.J.T., M.L., M.G.H., J.C.B., H.H., M.M.R.), UCL Institute of Neurology, Queen Square, London, UK; Department of Human Genetics and Hussman Institute for Human Genomics (M.A.G., S.Z.), Miller School of Medicine, University of Miami; The Genesis Project Foundation (M.A.G.), Miami, FL; The Dubowitz Neuromuscular Centre (F.M., A.Y.M.), UCL Institute of Child Health, London; and Department of Clinical Neurophysiology (J.C.B.), Norfolk and Norwich University Hospital, Norwich, UK.

## Abstract

**Objective:**

To describe the genetic and clinical features of a simplex patient with distal hereditary motor neuropathy (dHMN) and lower limb spasticity (Silver-like syndrome) due to a mutation in the sigma nonopioid intracellular receptor–1 gene (*SIGMAR1*) and review the phenotypic spectrum of mutations in this gene.

**Methods:**

We used whole-exome sequencing to investigate the proband. The variants of interest were investigated for segregation in the family using Sanger sequencing. Subsequently, a larger cohort of 16 unrelated dHMN patients was specifically screened for *SIGMAR1* mutations.

**Results:**

In the proband, we identified a homozygous missense variant (c.194T>A, p.Leu65Gln) in exon 2 of *SIGMAR1* as the probable causative mutation. Pathogenicity is supported by evolutionary conservation, in silico analyses, and the strong phenotypic similarities with previously reported cases carrying coding sequence mutations in *SIGMAR1*. No other mutations were identified in 16 additional patients with dHMN.

**Conclusions:**

We suggest that coding sequence mutations in *SIGMAR1* present clinically with a combination of dHMN and pyramidal tract signs, with or without spasticity, in the lower limbs. Preferential involvement of extensor muscles of the upper limbs may be a distinctive feature of the disease. These observations should be confirmed in future studies.

The distal hereditary motor neuropathies (dHMN) comprise a heterogeneous group of diseases that share the common feature of slowly progressive, symmetrical, and distal-predominant neurogenic weakness and amyotrophy.^1^ There is phenotypic and genetic overlap between dHMN and other hereditary neuropathies and motor neuron disorders such as axonal forms of Charcot-Marie-Tooth disease (CMT2), juvenile amyotrophic lateral sclerosis (ALS), and hereditary spastic paraplegia (HSP). *BSCL2*- and *REEP1*-related disorders are an example of this overlap as autosomal dominant mutations in these genes can cause dHMN with upper limb predominance, pure HSP, or spastic paraplegia with amyotrophy of hands (Silver syndrome) or hands and feet (table 1).^2–6^

**Table 1:**
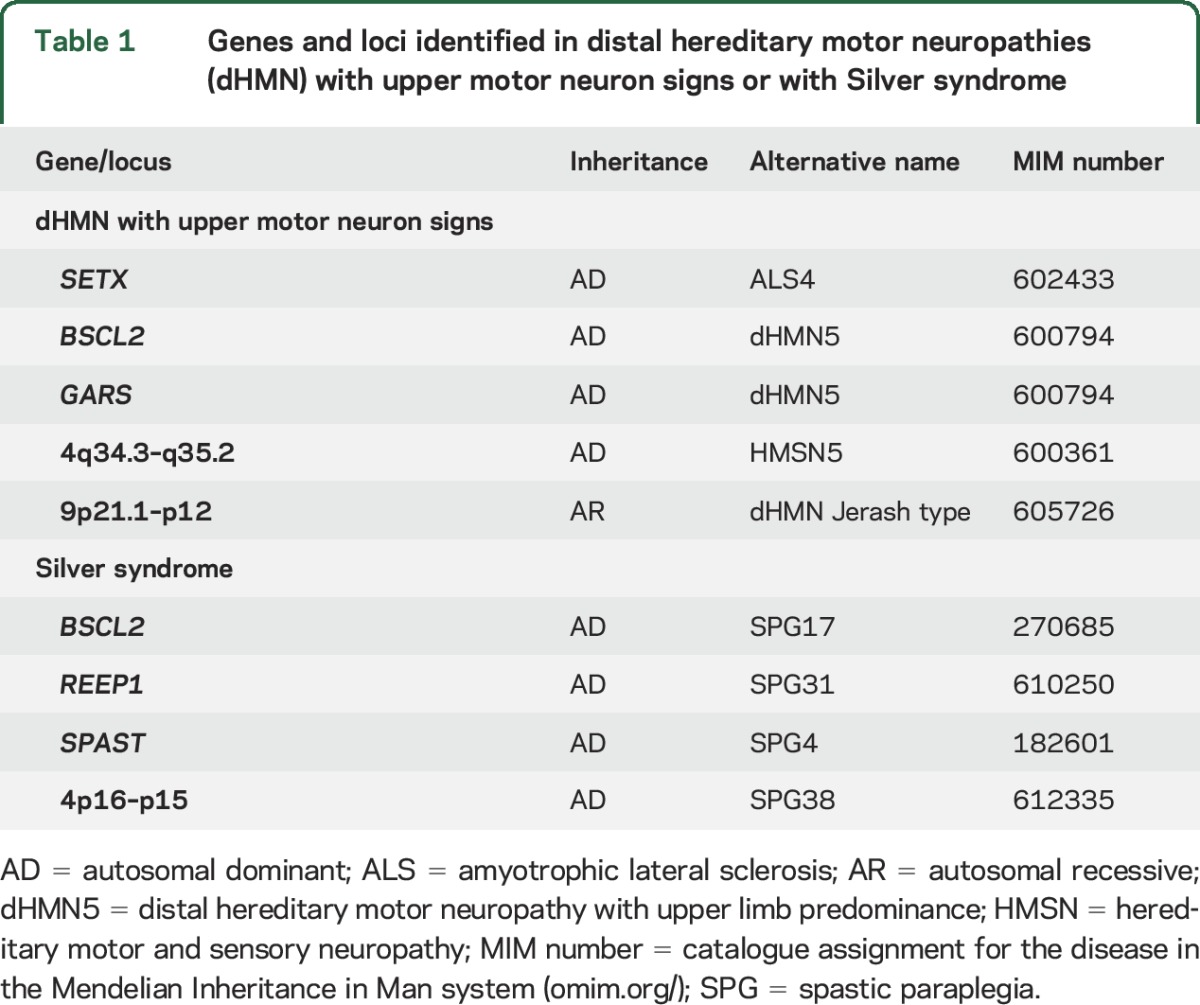
Genes and loci identified in distal hereditary motor neuropathies (dHMN) with upper motor neuron signs or with Silver syndrome

Homozygous mutations in the sigma nonopioid intracellular receptor-1 gene (*SIGMAR1*) have been reported as a cause of dHMN and juvenile ALS.^7,8^ Here we describe a patient with a Silver-like syndrome carrying a homozygous mutation in *SIGMAR1* and review the phenotypic spectrum of mutations in this gene. We suggest that coding sequence mutations present clinically with a combination of dHMN and pyramidal tract signs, with or without spasticity, in the lower limbs, adding *SIGMAR1* to the list of genes that can cause overlapping motor neuron/nerve phenotypes.

## METHODS

The proband and 16 additional patients screened for *SIGMAR1* mutations were identified from those attending neuropathy clinics at the National Hospital for Neurology and Neurosurgery, Queen Square, London, UK. The proband was investigated as part of a larger study to determine the genetic etiology in patients with inherited neuropathies using whole-exome sequencing (WES). All patients had undergone clinical and instrumental assessments during the routine diagnostic process and mutations in common neuropathy-related genes had been excluded in all cases. Nerve conduction studies, EMG, laboratory tests, and MRI scans were performed using standard methods. WES, Sanger sequencing, and in silico analysis methods are detailed in the supplementary material at Neurology.org.

### Standard protocol approvals, registration, and patient consents.

The study had ethical approval from the National Hospital for Neurology & Neurosurgery/Institute of Neurology Joint Research Ethics Committee. All patients gave written informed consent for genetic testing.

## RESULTS

### Clinical features.

The proband (figure, A) was the second child of healthy, nonconsanguineous parents of British and French descent. He walked independently at age 12 months. At age 3 years, he was noted to have bilateral foot drop and frequent falls and, over the following years, he developed progressive muscle weakness and atrophy in the lower limbs. Hand weakness and lower limb stiffness developed during the second decade of life. He underwent orthopedic surgery on his feet at ages 13–14 years and tendon-transfer surgery on both thumbs at ages 16–17 years with good functional outcomes. He has normal intellect, no sensory symptoms, and no sphincter problems. His family history was unremarkable and clinical and electrophysiologic examination of the parents was normal.

**Figure:**
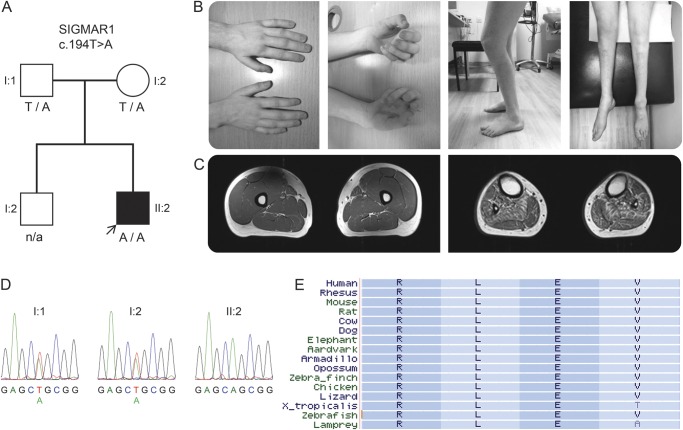
Family segregation, conservation of the *SIGMAR1* variant, and clinical images of the proband (A) Segregation of the *SIGMAR1* variant c.194T>A in the family; genotypes are indicated below tested individuals. (B) Photographs of the proband show atrophy of intrinsic hand muscles, clawed hands, flexed knee posture (characteristic of knee bobbing), and atrophy of leg and foot muscles. (C) Axial T1-weighted MRI of the mid-thighs (left) and mid-legs (right) demonstrate normal appearance of the thigh muscles and atrophy of all lower leg muscles with mild fatty replacement especially of tibialis anterior, tibialis posterior, soleus, and peroneal muscles. (D) Sanger sequencing electropherograms demonstrate sequence variants in the proband and his parents. (E) Conservation of leucine (L) at amino acid position 65 of the σ-1 receptor encoded by *SIGMAR1*; a subset of 16 species were chosen, representing the 100 species available at the USCS browser.

At age 17 years, on neurologic examination, he had clawed hands with no fixed contractures, bilateral finger and foot drop, knee bobbing, marked muscle atrophy from mid-forearms and knees down (figure, B), and weakness of wrist extension (Medical Research Council [MRC] grade 2/5 right, 4/5 left), finger extension (1/5), intrinsic hand muscles (0/5), ankle dorsiflexion (0/5 right, 1/5 left), ankle plantar flexion (1/5), toe extension (1/5), and toe flexion (0/5). Muscle strength in proximal muscles and finger flexors was normal. There was marked spasticity of the lower limbs and normal muscle tone in the upper limbs. Deep tendon reflexes were present in the upper limbs, increased at the knees, and absent at the ankles. Babinski sign was present bilaterally. Sensory and cranial nerve examinations were unremarkable. He walked with a combination of spastic and steppage gait. There was no scoliosis. Nerve conduction studies were consistent with a motor axonal neuropathy that was more severe in the lower limbs (table e-1). The superficial peroneal sensory action potential was normal and although the sural sensory nerve action potential amplitude was mildly reduced this may have been due to previous surgery rather than a sensory neuropathy. EMG of the biceps brachii showed features of chronic denervation. Brain and spinal cord MRIs were normal. Charcot-Marie-Tooth Neuropathy Score v.2 was 13.^9^

At age 20, neurologic examination confirmed progression of wrist extension weakness (MRC grade 1/5) causing hand drop and weakness of finger flexion (4/5) (video). The remainder of the examination was unchanged. MRI of the lower limbs demonstrated normal appearance of the thigh muscles and atrophy of all lower leg muscles with mild fatty replacement (figure, C). Serum creatine kinase level was normal at 184 IU/L.

### Genetic studies.

Molecular genetic analysis of the 17p11.2 chromosome region and direct sequencing of *MFN2*, *HSPB1*, *HSPB8*, *HSPB3*, *BSCL2,* and *GARS* genes revealed no pathogenic variants. WES on the proband revealed a total of 25,073 exonic variants (table e-2). The GEM.app software was used to focus on nonsynonymous, splice-site, and coding indel variants that were present in <5 families in the GEM.app database and that had a minor allele frequency (MAF) <0.5% in the Exome Variant Server database (http://evs.gs.washington.edu/EVS/). From a total of 198 variants that met these filtering criteria, 9 of them involved genes associated with pure and complex inherited neuropathies/neuronopathies. Six were heterozygous variants in genes associated with autosomal recessive disorders (*ERCC6*, *LAMA2*, *MTPAP*, *MTTP*, *NTRK1*, and *PRX*). Two heterozygous variants were detected in genes associated with autosomal dominant spinal muscular atrophy and CMT2 (*BICD2* and *TRPV4*) and one homozygous variant was detected in *SIGMAR1*, a gene associated with autosomal recessive dHMN and juvenile ALS. The variants detected in *BICD2*, *TRPV4*, and *SIGMAR1* were validated by direct sequencing.

Cosegregation analyses allowed exclusion of the *BICD2* and *TRPV4* variants as pathogenic (e-Results) and confirmed that the proband's parents were heterozygous carriers of the *SIGMAR1* variant c.194T>A located in exon 2 (Ensembl ID ENST00000277010) (figure, D). This variant was present in 4 out of 97,910 alleles (MAF 0.004%) in the Exome Aggregation Consortium database (ExAC http://exac.broadinstitute.org/), with none of the carriers being homozygous, and was absent in the GEM.app database and in an in-house exome database of 138 clinically and neuropathologically normal controls. c.194T>A leads to the substitution of nonpolar leucine for polar glutamine at amino acid position 65 (p.Leu65Gln), affects a highly conserved nucleotide and amino acid (figure, E), and is predicted as being deleterious or disease-causing by pathogenicity prediction tools (table e-3).

The 4 coding exons, intron/exon boundaries, and the 3′-untranslated region (3′-UTR) of *SIGMAR1* were Sanger sequenced in the proband and 16 additional unrelated dHMN patients. No other exonic or intronic changes were identified (table e-4).

## DISCUSSION

Using WES in a simplex patient with distal weakness and amyotrophy and lower limb spasticity and electrophysiologic features consistent with a motor neuropathy, we have identified *SIGMAR1* as the probable causative gene. The phenotype of the patient mimics classical Silver syndrome except for the absence of upper limb predominance and the autosomal recessive pattern of inheritance; we termed this novel phenotype Silver-like syndrome. Pathogenicity of the *SIGMAR1* variant is supported by the in silico analysis described above and previous reports of individuals carrying homozygous mutations in the same gene, which are summarized in table 2.^7,8^

**Table 2:**
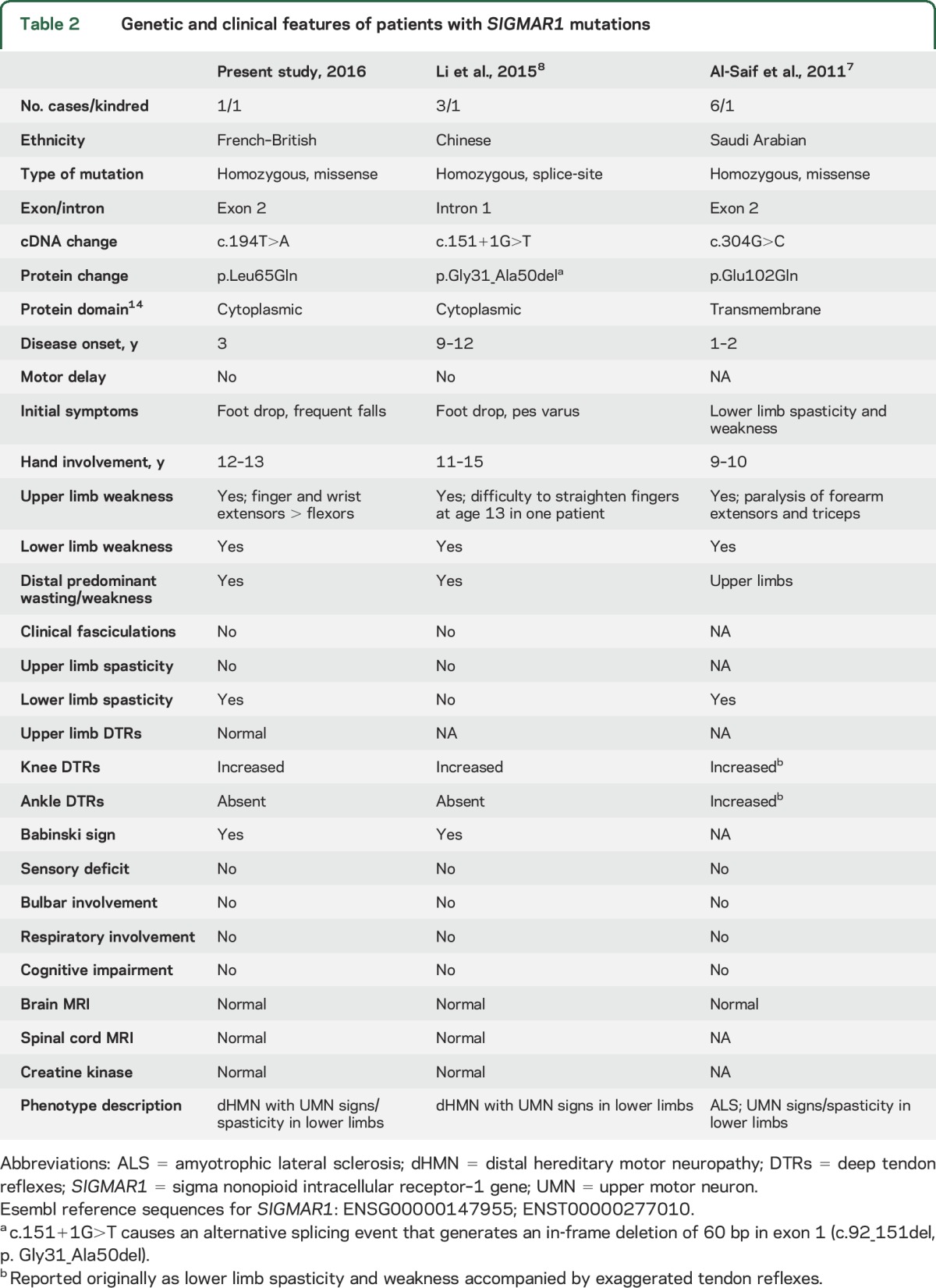
Genetic and clinical features of patients with *SIGMAR1* mutations

Like the present case, affected individuals from previously reported families developed a combination of distal weakness in the upper ± lower limbs and pyramidal features in the lower limbs. Both our case and those with the mutation c.304G>C, located in exon 2, presented within the first 3 years of life and had severe involvement of forearm extensors and lower limb spasticity.^7^ Although the authors designated the cases with c.304G>C as juvenile ALS, they had no bulbar involvement and EMG evidence of active denervation is not mentioned in the report. Thus, it appears that all these patients with exon 2 mutations share the same phenotype.

The cases with dHMN due to the splice-site mutation c.151+1G>T, resulting in deletion of the last 20 amino acids encoded by exon 1, had a later age at onset and lacked spasticity.^8^ As suggested by the authors, this phenotype is similar to that described in patients with dHMN and pyramidal features identified in the Jerash region of Jordan (dHMN-J) and mapped to 9p21.1-p12, which encompasses *SIGMAR1*.^10^ It is also significant that, as observed in our patient and those with the mutation c.304G>C, patients with dHMN-J had preferential involvement of forearm extensors over flexors.

The homozygous variant rs4879809 (9:34635598T>C, c.*31A>G), located in the 3′-UTR of *SIGMAR1*, has been detected in a consanguineous family with adult-onset ALS.^11^ The authors suggested that c.*31A>G could contribute to the pathogenesis of ALS in this family. The allele C in 9:34635598, however, has a frequency of 99.5% in the ExAC database (99% homozygotes) and was present in all patients from our cohort and the proband's unaffected parents so we regarded it as nonpathogenic. Heterozygous 3′-UTR variants in *SIGMAR1* have been also identified in individuals with frontotemporal lobar degeneration–motor neuron disease but their pathogenic role is still unclear.^12,13^

*SIGMAR1* encodes for the sigma-1 receptor (σ_1_R), an endoplasmic reticulum (ER) chaperone that resides at the specialized mitochondrial-associated ER membrane.^14^ σ_1_R is involved in a wide array of cellular processes, including ER-mitochondrial Ca^2+^ signaling and cell survival, and is highly expressed in motor neurons of the brainstem and spinal cord.^14,15^ Recent studies suggest that loss of σ_1_R function causes ER–mitochondria disconnection and ER stress activation and disrupts mitochondrial function and axonal transport, leading to motor neuron axonal degeneration followed by cell death.^16^ This dying-back degeneration process would be consistent with the distal predominant pattern of motor involvement seen in patients with *SIGMAR1* mutations.

Based on the present study and previous reports, we propose that coding sequence mutations in *SIGMAR1* manifest clinically with a combination of dHMN and pyramidal tract signs, with or without spasticity, in the lower limbs, and that preferential involvement of forearm extensors may be a distinctive feature of the disease. These observations should be confirmed in future studies.

## Supplementary Material

Data Supplement

Video

## GLOSSARY

ALS: amyotrophic lateral sclerosis
CMT: Charcot-Marie-Tooth disease
dHMN: distal hereditary motor neuropathy
dHMN-J: distal hereditary motor neuropathy and pyramidal features identified in the Jerash region of Jordan
ER: endoplasmic reticulum
ExAC: Exome Aggregation Consortium database
HSP: hereditary spastic paraplegia
MAF: minor allele frequency
MRC: Medical Research Council
σ1R: sigma-1 receptor
SIGMAR1: sigma nonopioid intracellular receptor–1 gene
UTR: untranslated region
WES: whole-exome sequencing
