# Supplementary material for: SIGMAR1 mutation associated with autosomal recessive Silver-like syndrome
Source: Neurology. 2016 Oct 11;87(15):1607–12. doi: 10.1212/WNL.0000000000003212 (PMC5067545; doi:10.1212/WNL.0000000000003212)
Supplement: Data Supplement [file supp_87_15_1607__index.html]

SIGMAR1 mutation associated with autosomal recessive Silver-like syndrome — Data Supplement 

# *SIGMAR1* mutation associated with autosomal recessive Silver-like syndrome

## Data Supplement

**Neurology® data supplements are not copyedited before publication. Published editorials and translations have been copyedited.  
 © 2016 American Academy of Neurology.  
  
 Files in this Data Supplement:**

- Data Supplement - PDF
