# Supplementary material for: SIGMAR1 mutation associated with autosomal recessive Silver-like syndrome
Source: Neurology. 2016 Oct 11;87(15):1607–12. doi: 10.1212/WNL.0000000000003212 (PMC5067545; doi:10.1212/WNL.0000000000003212)
Supplement: Data Supplement [file supp_WNL.0000000000003212_Supplementary_Material.pdf]

Supplementary material

SIGMAR1 mutation associated with autosomal recessive Silver-like syndrome

Alejandro Horga,[1] Pedro J Tomaselli,[1] Michael A Gonzalez,[2,3] Matilde Laurà,[1] Francesco Muntoni,[4] Adnan Y Manzur,[4] Michael G Hanna,[1] Julian C Blake,[1,5] Henry Houlden,[1] Stephan Züchner,[2] Mary M Reilly[1]

[1]MRC Centre for Neuromuscular Diseases, UCL Institute of Neurology, Queen Square, London, UK. [2]Department of Human Genetics and Hussman Institute for Human Genomics, Miller School of Medicine, University of Miami, Miami, Fl, USA. [3]The Genesis Project Foundation, Miami, Fl, USA. [4]The Dubowitz Neuromuscular Centre, UCL Institute of Child Health, London, UK. [5]Department of Clinical Neurophysiology, Norfolk and Norwich University Hospital, Norwich, UK.

Corresponding author: Professor Mary M. Reilly (email: m.reilly@ucl.ac.uk)

This file includes:

Tables e-1, e-2, e-3 and e-4

e-Methods

e-Results

e-References

Tables e-1, e-2, e-3 and e-4

Table e-1. Nerve conduction studies in the proband at age 17 years

	CMAP, mV	MNCV, m/s	DML, ms	SNAP, μV	SNCV, m/s
**Median nerve**	2.3	45	4.2	18	54
**Ulnar nerve**	0.9	40	3.3	15	58
**Radial nerve**	-	-	-	49	69
**Common peroneal nerve**	0.4	32	4.7	-	-
**Superficial peroneal nerve**	-	-	-	18	46
**Posterior tibial nerve**	0.2	-	4.7	-	-
**Sural nerve**	-	-	-	8	44

CMAP = compound muscle action potential; MNCV = motor nerve conduction velocity; DML = distal motor latency; SNAP = sensory nerve action potential; SNCV = sensory nerve conduction velocity.

**Table e-2. Whole-exome sequencing results and variant filtering**	
**Sequencing platform**	Illumina HiSeq 2000
**Target enrichment system**	Agilent SureSelect Human All Exon v5
**Alignment tool**	Burrows-Wheeler Aligner v0.7.12
**Variant calling tool**	FreeBayes 0.9.24
**Total no. of reads**	68,455,509
**30x coverage**	60.5%
**20x coverage**	69.9%
**10x coverage**	82.3%
**2x coverage**	95.3%
**Total variants**	102,218
Exonic variants	25,073
MAF <0.5% in EVS	3,705
In <5 pedigrees in GEM.app	198
In known neuropathy-related genes	9
Consistent with phenotype / inheritance	1

MAF = minor allele frequency; EVS = Exome Variant Server (http://evs.gs.washington.edu/); GEM.app = Genome Management Application (https://genomics.med.miami.edu/).

Table e-3. In silico analysis of the *SIGMAR1* variant c.194T>A; p.Leu65Gln

	**Score**	**Interpretation**
**Grantham (Leu, Gln)**	113	Moderate physicochemical difference
**PhyloP**	4.51	Highly conserved nucleotide
**GERP**	4.8	Highly conserved nucleotide
**SIFT**	0.000	Damaging
**PROVEAN**	-5.10	Deleterious
**PolyPhen-2 (HumVar)**	0.998	Probably damaging
**Mutation Taster**	Disease causing, p. 0.999	Disease causing, high probability
**Align-GVGD**	Class C65	Likely to interfere with protein function
**SuSPect**	80	Disease causing
**MutPred**	0.87	Probably deleterious[a]
**CADD**	22.5	Probably deleterious[b]

Grantham = Grantham Score (supplementary reference 7); PhyloP = PhyloP basewise conservation score derived from multiple sequence alignment of 46 vertebrate species (https://genome.ucsc.edu/); GERP = Genomic Evolutionary Rate Profiling score (https://genome.ucsc.edu/); SIFT = Sorting Intolerant From Tolerant algorithm (http://sift.jcvi.org/); PROVEAN = Protein Variation Effect Analyzer algorithm (http://www.mutationtaster.org/); PolyPhen2 = Polymorphism Phenotyping v2 software (http://genetics.bwh.harvard.edu/pph2/); Mutation Taster = Mutation Taster v2 software (http://www.mutationtaster.org/); Align-GVGD = Align-GVGD software (http://agvgd.iarc.fr/); SuSPect = Disease-Susceptibility-based SAV Phenotype Prediction tool (http://www.sbg.bio.ic.ac.uk/~suspect/); MutPred = MutPred computational model (http://mutpred.mutdb.org/); CADD = Combined Annotation Dependent Depletion tool (http://cadd.gs.washington.edu/).

a) Probability of deleterious mutation = 87%; b) Predicted to be within the 1% most deleterious substitutions in the human genome.

Table e-4. *SIGMAR1* sequence variants detected in patients with dHMN (n = 17)

rs ID	gDNA change GRCh37/hg19	cDNA change	Number of patients Het.	Hom.	MAF 1000G	MAF EVS	MAF ExAC
rs111496127	9:34637750C>G	c.-53G>C	1	0	n/r	n/r	n/r
rs140376902	9:34637375A>T	c.194T>A	0	1	n/r	0.008%	0.004%
rs4879809	9:34635598T>C	c.*31A>G	0	17	98.74%[a]	98.65%[a]	99.49%[a]
rs188221803	9:34635587C>A	c.*42G>T	0	1	0.08%	0.131%	0.08%
rs41305341	9:34635337T>G	c.*292A>C	2	1	3,43%	n/r	n/r

Esembl reference sequences for *SIGMAR1*: ENSG00000147955; ENST00000277010. rs ID = reference single nucleotide polymorphism identifier; Het. = heterozygous; Hom = homozygous; MAF = minor allele frequency; 1000G = 1000 Genomes Project (http://browser.1000genomes.org/); EVS = Exome Variant Server (http://evs.gs.washington.edu/); ExAC = Exome Aggregation Consortium (http://exac.broadinstitute.org/); N/R = not reported.

a) Frequency of the allele C.

e-Methods

Sanger sequencing. The coding regions and flanking intronic regions of *SIGMAR1* (Ensembl reference sequences: ENSG00000147955; ENST00000277010) were PCR-amplified using FastStart PCR Master Mix (Roche). Primer sequences and PCR conditions are available upon request. PCR products were cleaned up using the ExoSAP-IT treatment (Affymetrix). Sequencing reactions were performed using Big Dye Terminator v3.1 Cycle Sequencing Kit (Applied Biosystems) and products were purified using the Sephadex G50 filtration kit (Thermo Scientific ABgene). DNA fragments were separated on an ABI3730XL automatic DNA sequencer (Applied Biosystems). The resulting sequences were analysed with SeqScape software v2.5 (Applied Biosystems).

Whole-exome sequencing. The exomes of the proband were enriched using the SureSelect Human All Exon V5 capture kit (Agilent) and sequenced on the HiSeq 2000 platform (Illumina) at the John P. Hussman Institute for Human Genomics, University of Miami Miller School of Medicine, Miami, FL, USA. The resulting 100bp paired-end sequence reads were mapped against the human reference genome assembly 19 (GRCh37) with the Burrows-Wheeler Aligner (BWA) package.[e1] Variant calling and indel realignment were performed with FreeBayes.[e2] All variants were submitted to SeattleSeq for annotation (http://snp.gs.washington.edu/).[e3] VCF files were imported to GEM.app for further analysis and candidate variant identification (table e-1).[e4]

Bioinformatic analysis. cDNA and protein sequence variants are described in accordance with the recommendations of the Human Genome Variation Society (http://www.hgvs.org) using Ensembl ENSG00000147955; ENST00000277010 as the reference sequences (http://www.ensembl.org/). Evolutionary conservation of nucleotides was assessed using PhyloP (46 vertebrate species) and GERP

scores,[e5,e6] which were accessed through the UCSC Genome Browser (https://genome.ucsc.edu/) using genomic coordinates from GRCh37/hg19. Grantham scores were used to assess the physicochemical nature of the amino acid substitutions.[e7] In silico analyses of sequence variants were performed using the following pathogenicity prediction tools: SIFT v.1.03 (http://sift.jcvi.org/),[e8] PROVEAN v.1.1.3 (http://sift.jcvi.org/),[e9] PolyPhen-2 (http://genetics.bwh.harvard.edu/pph2/),[e10] Mutation Taster v.2 (http://www.mutationtaster.org/);[e11] Align-GVGD (http://agvgd.iarc.fr/),[e12] SuSPect (http://www.sbg.bio.ic.ac.uk/~suspect/);[e13] MutPred (http://mutpred.mutdb.org/)[e14] and CADD (http://cadd.gs.washington.edu/).[e15]

e-Results

***BICD2* and *TRPV2* variants**. Segregation analyses revealed that the *BICD2* variant c.2142G>C (p.Lys714Asn; rs777986224), with a MAF of 0.003% in the Exome Aggregation Consortium database (ExAC; http://exac.broadinstitute.org/), was present in the unaffected father of the proband, and that the *TRPV4* variant c.1378C>T (p.Arg460Trp; rs34227547), with a MAF of 0.01% in the ExAC database, was present in the unaffected mother. Thus, these two variants were regarded as non-pathogenic.

e-References

e1. Li H, Durbin R. Fast and accurate short read alignment with Burrows-Wheeler transform. Bioinformatics 2009;25:1754-60.

e2. Garrison E, Marth G. Haplotype-based variant detection from short-read sequencing. *arXiv preprint arXiv:1207.3907 [q-bio.GN]* 2012.

e3. Ng SB, Turner EH, Robertson PD, et al. Targeted capture and massively parallel sequencing of 12 human exomes. Nature 2009;461:272-6.

e4. Gonzalez MA, Lebrigio RF, Van Booven D, et al. GEnomes Management Application (GEM.app): a new software tool for large-scale collaborative genome analysis. Hum Mutat 2013;34:842-6.

e5. Pollard KS, Hubisz MJ, Rosenbloom KR, Siepel A. Detection of nonneutral substitution rates on mammalian phylogenies. Genome Res 2010;20:110-21.

e6. Cooper GM, Stone EA, Asimenos G, Green ED, Batzoglou S, Sidow A. Distribution and intensity of constraint in mammalian genomic sequence. Genome Res 2005;15:901-13.

e7. Grantham R. Amino acid difference formula to help explain protein evolution. Science 1974;185:862-4.

e8. Ng PC, Henikoff S. SIFT: Predicting amino acid changes that affect protein function. Nucleic Acids Res 2003;31:3812-4.

e9. Choi Y, Sims GE, Murphy S, Miller JR, Chan AP. Predicting the functional effect of amino acid substitutions and indels. PLoS ONE 2012;7:e46688.

e10. Adzhubei IA, Schmidt S, Peshkin L, et al. A method and server for predicting damaging missense mutations. Nat Methods 2010;7:248-9.

e11. Schwarz JM, Cooper DN, Schuelke M, Seelow D. MutationTaster2: mutation prediction for the deep-sequencing age. Nat Methods 2014;11:361-2.

e12. Tavtigian SV, Deffenbaugh AM, Yin L, et al. Comprehensive statistical study of 452 BRCA1 missense substitutions with classification of eight recurrent substitutions as neutral. J Med Genet 2006;43:295-305.

e13. Yates CM, Filippis I, Kelley LA, Sternberg MJ. SuSPect: enhanced prediction of single amino acid variant (SAV) phenotype using network features. J Mol Biol 2014;426:2692-701.

e14. Li B, Krishnan VG, Mort ME, et al. Automated inference of molecular mechanisms of disease from amino acid substitutions. Bioinformatics 2009;25:2744-50.

e15. Kircher M, Witten DM, Jain P, O'Roak BJ, Cooper GM, Shendure J. A general framework for estimating the relative pathogenicity of human genetic variants. Nat Genet 2014;46:310-5.
