# Supplementary material for: SIGMAR1 mutation associated with autosomal recessive Silver-like syndrome
Source: Neurology. 2016 Oct 11;87(15):1607–12. doi: 10.1212/WNL.0000000000003212 (PMC5067545; doi:10.1212/WNL.0000000000003212)
Supplement: Video [file supp_WNL.0000000000003212_Video_Legend.docx]

**Video Legend:** Video of patient at 20 years of age demonstrating spastic gait with bilateral foot drop, weakness of finger and wrist extension, and atrophy of hand and lower leg muscles.
